# Supplementary figures and images for: External task switches activate default mode regions without enhanced processing of the surrounding scene
Source: Imaging Neurosci (Camb). 2024 Jun 3;2:imag-2-00185. doi: 10.1162/imag_a_00185 (PMC12247583; doi:10.1162/imag_a_00185)

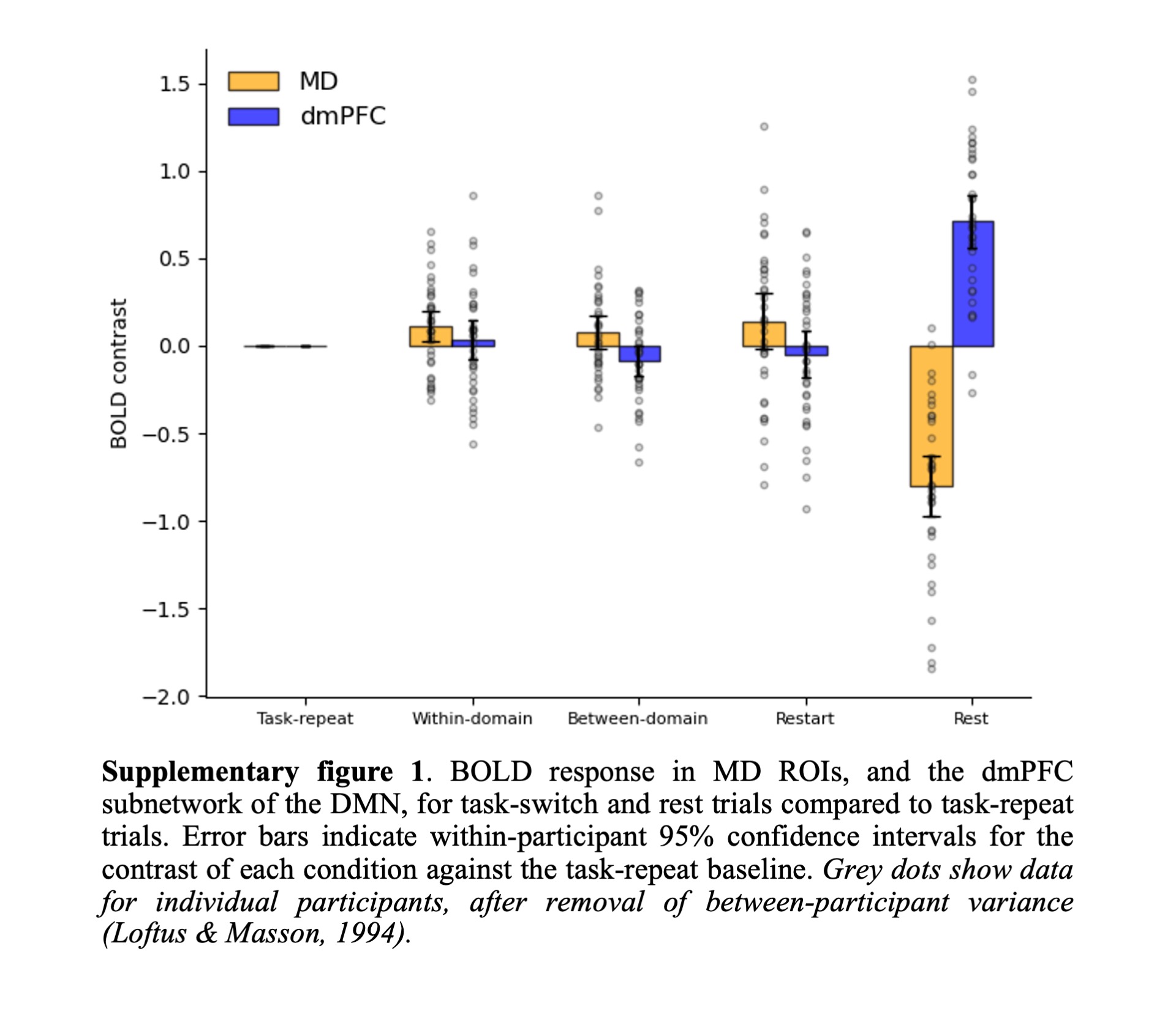

Supplement: Supplementary Material [file imag_a_00185-supp.jpeg]
